# Annual acknowledgement of manuscript reviewers

**DOI:** 10.1186/1742-4682-12-4

**Published:** 2015-07-03

**Authors:** Hiroshi Nishiura, Edward Rietman, Rongling Wu

**Affiliations:** The University of Tokyo, Tokyo, Japan; Tufts University School of Medicine, Boston, USA; Pennsylvania State University, State College, USA

## Abstract

The Editors of Theoretical Biology and Medical Modelling would like to thank all the Reviewers who have contributed to the journal in Volume 11 (2014).

**Paul Agutter**

United Kingdom

**Gary An**

United States of America

**Andrea Apolloni**

United Kingdom

**Nicolas Bacaer**

France

**Bo Baldetorp**

Sweden

**Amy Bauer**

United States of America

**Deepak Bharkhada**

United States of America

**Donald Buerk**

United States of America

**Ted Carmichael**

United States of America

**Nilay Chakraborty**

United States of America

**Xiao Chang**

United States of America

**Hamidreza Chitsaz**

United States of America

**Thanat Chookajorn**

Thailand

**Gerardo Chowell**

United States of America

**Chaeshin Chu**

Korea, South

**Jean Clairambault**

France

**Vittoria Colizza**

France

**Giuseppe Colloca**

Italy

**Michel Costa**

Brazil

**Gheorghe Craciun**

United States of America

**Damian Craiem**

Argentina

**Marc Antoine Custaud**

France

**Petr Danecek**

United Kingdom

**Kiranmoy Das**

United States of America

**Bo Ding**

United States of America

**Yueping Dong**

Japan

**Ilaria Dorigatti**

United Kingdom

**Michael Ederer**

Germany

**Keisuke Ejima**

Japan

**Gloria Elliott**

United States of America

**Heiko Enderling**

United States of America

**Ivo Foppa**

United States of America

**Holly Freedman**

Canada

**Marc Garbey**

United States of America

**Stephen Grand**

United States of America

**Xueying Guan**

United States of America

**Mohammed Guedda**

France

**Shakti Gupta**

United States of America

**Konstantin Gurevich**

Russian Federation

**Robert G Hahn**

Sweden

**Ulrich Hartmann**

Germany

**Sereina Herzog**

Austria

**Atsushi Hijikata**

Japan

**Yoshito Hirata**

Japan

**Sharon Hori**

United States of America

**Tingjun Hou**

China

**Tao Huang**

China

**Marcos Intaglietta**

United States of America

**Shuji Ishihara**

Japan

**Charles William Jefford**

Switzerland

**Milan Jokanovic**

Serbia

**Antonio Juretic**

Croatia

**Irina Kareva**

United States of America

**Ruian Ke**

United States of America

**Claude Krummenacher**

United States of America

**Olga Krylova**

Canada

**Chris Leary**

United States of America

**Marie-Paule Lefranc**

France

**Huan Lei**

United States of America

**Xuejin Li**

United States of America

**Zhengfei Lu**

United States of America

**Feilim Mac Gabhann**

United States of America

**Dann Mallet**

Australia

**Natalia Mantilla-Beniers**

Mexico

**Peter Mazur**

United States of America

**James McCaw**

Australia

**Attila Miseta**

Hungary

**Takashi Miura**

Japan

**Kenji Mizumoto**

Japan

**Shinji Nakaoka**

Japan

**Satyaprakash Nayak**

United States of America

**Hiroshi Nishiura**

Japan

**Ae Nim Pae**

Korea, South

**Klot Patanarapeelert**

Thailand

**Maria Angeles Perez**

Spain

**Marco Pettini**

France

**Arkady Pikovsky**

Germany

**David Regan**

Australia

**Xianwen Ren**

China

**Edward Rietman**

United States of America

**Jean Rodolphe Roche**

France

**Wagner Rodrigo Weinert**

Brazil

**Libin Rong**

United States of America

**Kamlesh Sahu**

Canada

**Mahdi Sanati**

United States of America

**Jean Sanders**

United States of America

**Fumitoshi Sato**

Japan

**Nick Savill**

United Kingdom

**Daniel Schmidt**

Australia

**Jan Schmoranzer**

Germany

**Jim Slattery**

United Kingdom

**Gerhard Smekal**

Austria

**Silvia Soddu**

Italy

**William Spillman**

United States of America

**Andreas Steingoetter**

Switzerland

**Kaoru Sugimura**

Japan

**Akos Sveiczer**

Hungary

**Michael Takaza**

Ireland

**Jijun Tang**

United States of America

**Daniel Tartakovsky**

United States of America

**Yu Teng**

United States of America

**David Thomas**

United States of America

**Tianhai Tian**

Australia

**Jack Tuszynski**

Canada

**Gert Verpooten**

Belgium

**BOUARIFI Walid**

Morocco

**Guanyu Wang**

United States of America

**Zhilin Wang**

China

**Yaqun Wang**

United States of America

**Tarynn Witten**

United States of America

**Song Wu**

United States of America

**Jianhong Wu**

Canada

**Si Wu**

China

**Dan Xue**

China

**Naji Yebari**

Morocco

**Yao Yu**

United States of America

**Yongyuth Yuthavong**

Thailand

**Tao Zhang**

China

**Ni Zhao**

United States of America

